# Age-specific associations of RBC folate and several serum folate forms with obesity risk: NHANES 2011–2018

**DOI:** 10.3389/fnut.2025.1547844

**Published:** 2025-04-10

**Authors:** Meng Wang, Zonghang Tong, Chaoxue Li, Yanhong Wang, Xueli Yang, Zhongying Gong, Qiang Zhang, Xuan Wang, Xumei Zhang

**Affiliations:** ^1^Department of Nutrition and Food Science, School of Public Health, Tianjin Medical University, Tianjin, China; ^2^Tianjin Key Laboratory of Environment, Nutrition and Public Health, Center for International Collaborative Research on Environment, Nutrition and Public Health, Tianjin Medical University, Tianjin, China; ^3^Key Laboratory of Prevention and Control of Major Diseases in the Population, Ministry of Education, Tianjin Medical University, Tianjin, China; ^4^Affiliated Hospital of Changzhi Institute of TCM, Changzhi, Shanxi, China; ^5^Department of Occupational and Environmental Health, School of Public Health, Tianjin Medical University, Tianjin, China; ^6^Department of Neurology, Tianjin First Center Hospital, Tianjin, China

**Keywords:** RBC folate, 5-methyltetrahydrofolate, unmetabolized folic acid, obesity, NHANES

## Abstract

**Background:**

This study aims to explore the associations between RBC folate, several serum folate forms [serum total folate, 5-methyltetrahydrofolate (5-mTHF), and unmetabolized folic acid (UMFA)], and obesity risk in middle-aged and older populations.

**Methods:**

Data from NHANES (2011–2018) included 11,615 participants. Generalized linear models (GLMs) were applied to investigate associations of RBC folate and various serum folate forms with obesity risk after multivariable adjustment. Potential effect modifications were examined through stratified analyses and multiplicative interaction testing.

**Results:**

Among the total sample, middle-aged, and older participants, 4578 (39.4%), 3613 (40.0%), and 965 (37.2%) were obese, respectively. A positive association between RBC folate and obesity risk was observed, with the highest risks of obesity were consistently found in the fourth quartile (≥ 1,430 nmol/L) for the middle-aged adults (OR = 1.104, 95% CI: 1.045–1.166) and the older participants (OR = 1.157, 95% CI: 1.036–1.293). A significant negative association between serum total folate levels and obesity risk in middle-aged participants, with an OR of 0.804 (95% CI: 0.773–0.835) in the highest quartile (≥ 54.2 nmol/L). Similarly, serum 5-mTHF levels were negatively associated with obesity risk, with an OR of 0.800 (95% CI: 0.772–0.830) in the highest quartile (≥ 51.2 nmol/L). Most importantly, older participants with UMFA levels in the fourth quartile (≥ 1.06 nmol/L) had a higher risk of obesity (OR, 1.056; 95% CI: 1.004–1.110) compared with those with lower UMFA levels, but this association was not found in the total participants or the middle-aged participants.

**Conclusion:**

Significant positive relationships exist between RBC folate and obesity risk. Additionally, low serum 5-mTHF in middle-aged participants and high UMFA in older adults were associated with increased obesity risk, highlighting the importance of monitoring folate concentrations for guiding future clinical trials on folate supplementation.

## 1 Introduction

Obesity has become a growing public health problem worldwide, with its prevalence rising significantly over the past few decades (1). It is associated with an increased risk of various diseases, including cardiovascular disease, diabetes mellitus, certain cancers, and mental health disorders, all of which negatively impact quality of life, work productivity, and healthcare costs (2). As the global obesity epidemic continues to intensify, the need for effective prevention and treatment strategies has become a pressing priority in public health.

Recent studies have reported the associations between RBC folate, serum total folate levels and obesity, but the findings remain inconsistent. Some studies reported negative associations between obesity and total folate levels (3), while an analysis using NHANES data identified a positive association between obesity and erythrocyte folate in the overall population (4). In contrast, other studies found no significant relationship between serum total folate and body weight in older individuals (5). Therefore, conclusions on the as-sociation between RBC folate, serum total folate, and obesity remain inconclusive, and more importantly, the associations between RBC folate, serum total folate and obesity are not consistent across populations, making it crucial to consider age differences among groups.

Mandatory fortification and folic acid supplementation doubled the United Staes population’s serum folate concentration in the past 30 years. Different circulating folate forms may have diverse health impacts. A prospective cohort study revealed significant associations of raised 5-methyltetrahydrofolate (5-mTHF) and unmetabolized folic acid (UMFA) levels with increased mortality rates (6), while serum levels of 5-mTHF and UMFA had controversial effects on kidney dysfunction (7). It is crucial to deeply understand the effects of excessive folate intake from supplementation and fortified foods on health and diseases (8, 9). Further investigation is imperative to elucidate the influence of serum folate forms on health across diverse populations, accounting for the diverse array of folate types. Although several works have suggested risk of obesity linked to RBC folate and serum total folate in general populations (3, 10), it is worth comparing the potential differences between various serum folate forms and obesity in middle-aged and older populations, considering the metabolic changes with aging.

Using National Health and Nutrition Examination Surveys (NHANES) 2011–2018 data, this study is aimed to scrutinize associations of RBC folate and various serum folate forms (serum total folate, 5-mTHF, and UMFA) correlation with obesity, and to examine possible effect modifiers in United States adults.

## 2 Materials and methods

### 2.1 Study design and population

The NHANES, headed by the Centers for Disease Control and Prevention, collects United States children and adults’ nutritional and health data. It utilizes a complex multistage and probabilistic sampling method for a nationally representative sample (11). All documents pertaining to each survey are available on the NHANES website (12).

In this study, we analyzed the NHANES 2011 to 2018 data (*n* = 39,156), limiting the scope to non-pregnant persons over 20 (*n* = 22,370). We excluded 2,540 participants with missing serum or RBC folate information. From the 19,830 participants left, 7,817 were removed for missing confounder data, and 398 were taken off for having < 500 kcal/d or > 6,000 kcal/d energy intake. Hence, 11,615 subjects were finally included in our analysis (Supplementary Figure 1).

### 2.2 Measurements of RBC folate and serum folate forms

Serum and whole blood samples were drawn and analyzed at CDC’s Nutritional Biomarkers Lab. Field-collected specimens should be kept cool, light-protected, processed, frozen, and shipped overnight on dry ice, to store at ≤ −*20*°C until analysis. RBC folates were measured via a microbiologic assay. Five biologically active folate forms were performed on fresh or frozen serum without freeze thaw cycles using LC-MS/MS by the CDC laboratory (13). Details on specimen processing and laboratory methods have been described elsewhere (14). Long-term quality control CVs were < 3% for 5-mTHF, and mostly < 10% for other folate forms. Serum total folate was the sum of 5 active forms including MeFox (15). Imputed values [limit of detection (LOD) divided by square root of two] were used for any folate form result < LOD. If any folate concentration was absent, no serum total folate was calculated. The equations should be inserted in editable format from the equation editor.

### 2.3 Outcomes and covariation assessment

For measurement of obesity, trained health technicians measured body weight, height, and waist circumference at the mobile examination centers (MECs). BMI was calculated as weight (kg) divided by height squared (m^2^). General obesity was defined as BMI of 30 kg/m^2^ or higher, while overweight was defined as a BMI ranging from 25.0 to 29.9 kg/m^2^.

Demographic information, lifestyle, diet, and history of diseases were collected by direct interview through questionnaires. Smoking was defined as smoking at least 100 cigarettes in life. Drinking was defined as drinking at least 12 drinks a year. Hypertension was identified as systolic blood pressure ≥ 140 mmHg, diastolic blood pressure ≥ 90 mmHg, or self-reported diagnosis of hypertension. Diabetes mellitus was defined as a fasting blood glucose level ≥ 7.0 mmol/L, or a previous diagnosis of diabetes. Participants were asked about their physical activity during a typical week, based on the Global Physical Activity Questionnaire (GPAQ). Metabolic equivalent (MET) per week was calculated according to the GPAQ guideline. Participants without any PA and performing < 600 MET min/week were classified as inactive. Those performing ≥ 600 MET min/week were classified as active. Total energy intake per day, total fat intake per day, total sugars intake per day, food folate intake levels, and folate as dietary folate equivalents levels were based on dietary interview data, from which the total intake of the first 2 days was averaged as the participants’ intake. When data from the second day was absent, the data of the first day represented the typical total intake per day.

### 2.4 Statistical analysis

All statistical analyses accounted for complex survey design factors for NHANES, including sample weights, stratification, and clustering, following NHANES analytic and reporting guidelines (12). Comparison of baseline characteristics according to obesity status was performed by chi-square test for categorical variables and analysis of variance (ANOVA) for continuous variables.

Associations of obesity with RBC and other serum folate acids were examined in total participants, as well as in the middle-aged group (i.e., participants aged < 65 years) and in the older group (i.e., participants aged ≥ 65 years). Logistic regression models were used to estimate odds ratios (ORs) and 95% confidence intervals (CIs) for the associations according to quartiles of RBC folate or folate forms with the lowest quartile as the reference group. The crude model adjusted for age, sex (female, male), and ethnicity (Mexican American, Hispanic, non-Hispanic White, non-Hispanic Black, and others); Model 1 adjusted for age, sex, ethnicity, education level (Less than 9th grade, 9–11th grade, high school, some college or AA degree, college graduation, or above), marital status (Married, Widowed, Divorced, Separated, Never married, Living with partner), ratio of family income to poverty family (≤ 1.0, 1.0–3.0, > 3.0), physical activity status (active vs. inactive), waist circumference, total energy intake, total sugar intake, total fat intake, food folate intake, smoking status (yes vs. no), drinking status (yes vs. no), diabetes mellitus (yes vs. no), hypertension (yes vs. no); Model 2 adjusted for variables in model 1, plus mutually adjustment for the con-centration of other folate forms.

Stratification analyses were conducted to explore possible effect modifications of social and demographic variables including sex, smoking, alcohol use, diabetes, hypertension, and physical activity. We used logistic regression models to fit multiplicative interactions between two dichotomous variables.

A two-tailed *P* < 0.05 was statistically significant in all analyses. Analyses were performed using R 3.6.2 software^1^ and R package SURVEY.

## 3 Results

### 3.1 Baseline characteristics of the participants

Baseline characteristics of the study participants in total and by obesity status were presented in Table 1. Among the 11,615 adult participants, the prevalence of obesity was 39.4% (*n* = 4,578). The median (interquartile range, IQR) of age was 47.0 (33.0, 60.0) years, and 47.8% were men. The median (IQR) of RBC folate, serum total folate, 5-mTHF, and UMFA were 1120.0 (871.0, 1480.0) nmol/L, 38.7 (26.3, 55.3) nmol/L, 36.4 (24.5, 52.4) nmol/L, and 0.7 (0.51, 1.07) nmol/L, respectively. Table 1 also shows that obese ones were older and had higher proportions of women, compared to non-obese individuals. The obese participants reported less alcohol use, smoking, diabetes, and hypertension. They also had increased RBC folate and total fat intake but decreased serum total folate, 5-mTHF, and dietary folate equivalents.

**TABLE 1:** Characteristics of study population (*N* = 11,615), NHANES, United States, 2011–2018^†^.

Characteristics	Overall, *N* = 11,615^1^	No obesity, *N* = 7,037^1^	General obesity, *N* = 4,578^1^	*P* ^2^
Age, y	47.0 (33.0, 60.0)	46.0 (31.0, 60.0)	49.0 (36.0, 60.0)	< 0.001
**Gender, *n* (%)**				0.010
Male	5,568 (47.8%)	3,618 (49.3%)	1,950 (45.5%)	
Female	6,047 (52.2%)	3,419 (50.7%)	2,628 (54.5%)	
**Ethnicity, *n* (%)**				< 0.001
Mexican American	1,487 (8.5%)	775 (7.3%)	712 (10.4%)	
Other Hispanic	1,144 (5.4%)	673 (5.1%)	471 (6.0%)	
Non-Hispanic White	4,855 (67.4%)	2,989 (69.1%)	1,866 (64.8%)	
Non-Hispanic Black	2,482 (10.1%)	1,279 (8.3%)	1,203 (12.9%)	
Other ethnicity	1,647 (8.6%)	1,321 (10.2%)	326 (5.9%)	
**Education level, *n* (%)**				< 0.001
Less than 9th grade	787 (3.6%)	459 (3.4%)	328 (4.0%)	
9–11th grade	1,323 (8.3%)	771 (7.9%)	552 (9.1%)	
High school graduate	2,569 (21.6%)	1,442 (20.1%)	1,127 (24.2%)	
Some college or AA degree	3,726 (33.0%)	2,086 (30.5%)	1,640 (37.1%)	
College graduate or above	3,210 (33.4%)	2,279 (38.2%)	931 (25.6%)	
**Marital status, *n* (%)**				< 0.001
Married	6,010 (55.2%)	3,669 (55.3%)	2,341 (55.0%)	
Widowed	763 (4.9%)	438 (4.6%)	325 (5.5%)	
Divorced	1,290 (10.7%)	703 (9.3%)	587 (12.8%)	
Separated	382 (2.1%)	225 (2.0%)	157 (2.3%)	
Never married	2,224 (18.9%)	1,421 (20.5%)	803 (16.4%)	
Living with partner	946 (8.2%)	581 (8.3%)	365 (8.0%)	
**Physical activity, *n* (%)**				< 0.001
Active	8,306 (76.1%)	5,243 (80.4%)	3,063 (69.3%)	
Inactive	3,309 (23.9%)	1,794 (19.6%)	1,515 (30.7%)	
**PIR**				0.004
< 1	2,340 (13.7%)	1,351 (13.0%)	989 (15.0%)	
1∼3	4,805 (35.7%)	2,783 (34.4%)	2,022 (37.6%)	
≥ 3	4,470 (50.6%)	2,903 (52.6%)	1,567 (47.4%)	
BMI (kg/m^2^)	28.0 (24.3, 32.7)	25.3 (22.7, 27.5)	34.3 (31.8, 38.4)	< 0.001
Waist Circumference (cm)	98.2 (87.9, 109.7)	90.7 (83.0, 97.6)	113.0 (106.2, 122.0)	< 0.001
**Smoking, *n* (%)**				0.031
Ever	4,974 (42.6%)	2,960 (41.2%)	2,014 (44.8%)	
Never	6,641 (57.4%)	4,077 (58.8%)	2,564 (55.2%)	
**Alcohol use, *n* (%)**				0.002
Ever	8,848 (81.1%)	5,428 (82.5%)	3,420 (78.7%)	
Never	2,767 (18.9%)	1,609 (17.5%)	1,158 (21.3%)	
**Diabetes, *n* (%)**				< 0.001
Ever	1,582 (10.1%)	646 (5.8%)	936 (17.0%)	
Never	10,033 (89.9%)	6,391 (94.2%)	3,642 (83.0%)	
**Hypertension, *n* (%)**				< 0.001
Ever	4,202 (32.5%)	2,048 (25.2%)	2,154 (44.3%)	
Never	7,413 (67.5%)	4,989 (74.8%)	2,424 (55.7%)	
RBC folate (nmol/L)	1120.0 (871.0,1480.0)	1110.0 (854.0, 1440.0)	1150.0 (891.0, 1530.0)	< 0.001
Serum total folate (nmol/L)	38.7 (26.3, 55.3)	40.5 (27.9, 57.3)	35.6 (24.5, 51.5)	< 0.001
5-mTHF (nmol/L)	36.4 (24.5, 52.4)	38.4 (26.0, 54.3)	33.5 (22.7, 48.7)	< 0.001
UMFA (nmol/L)	0.70 (0.51, 1.07)	0.70 (0.51, 1.08)	0.70 (0.51, 1.06)	0.900
Total energy (kcal)	1985.0 (1570.5, 2521.5)	1995.5 (1580.0, 2507.5)	1976.5 (1549.0, 2532.0)	0.300
Total sugars (gm)	94.9 (63.7, 136.8)	95.5 (64.8, 136.5)	94.1 (62.3, 137.3)	0.500
Total fat (gm)	76.7 (56.9, 100.6)	76.0 (56.6, 98.6)	78.0 (57.3, 103.6)	0.037
Food folate (mcg)	201.5 (143.0, 277.5)	208.0 (149.5, 287.0)	189.0 (136.5, 264.5)	< 0.001
Folate, DFE (mcg)	464.0 (333.0, 653.5)	480.0 (345.0, 676.5)	442.5 (313.5, 618.5)	< 0.001

^†^Boldface indicates statistical significance (*P* < 0.05).

^1^Continuous values are given as the median (the 25% and 75% quartiles), and categorical variables are given as frequency (percentage).

^2^Chi-squared test with Rao and Scott’s second-order correction; Wilcoxon rank-sum test for complex survey samples. BMI, body mass index; PIR, ratio of family income to poverty; RBC folate, red blood cell folate; 5-mTHF, 5-methylenetetrahydrofolate; UMFA, unmetabolized folic acid.

### 3.2 Relationship of folate forms with the risk of obesity

After adjusting for covariates in Model 2, a significant positive association between RBC folate levels and obesity risk was observed with ORs of 1.053 (95% CI: 1.015–1.093), 1.073 (95% CI: 1.027–1.121), 1.105 (95% CI: 1.040–1.175) across the Q2, Q3, and Q4 groups, respectively (trend *P* < 0.001, Supplementary Table 1). This associations still existed within both the middle-aged group (quartile trend *P* < 0.001) (Table 2) and the older group (quartile trend *P* = 0.013) (Table 3), and the highest risks of obesity were consistently found in the top quartile (Q4) groups for the middle-aged adults (OR = 1.104, 95% CI: 1.045–1.166) and the older participants (OR = 1.157, 95% CI: 1.036–1.293).

**TABLE 2:** Associations between different folate forms and obesity in middle-aged participants^†^.

Variables	Total	Events (%)	Crude models^1^		Adjusted models 1^2^		Adjusted models 2^3^	
			**OR (95% CI)**	***P***	**OR (95% CI)**	***P***	**OR (95% CI)**	***P***
**RBC folate**	–	–	–	–	–	–	–	–
**Quartiles**	–	–	–	–	–	–	–	–
Q1 (< 824)	2,489	35.6	Reference	–	Reference	–	Reference	–
Q2 (824 < 1,090)	2,430	39.5	1.03 (0.99, 1.07)	0.158	1.05 (1.01, 1.09)	0.021	1.05 (1.01, 1.09)	0.012
Q3 (1,090 < 1,430)	2,296	39.9	1.02 (0.98, 1.07)	0.318	1.06 (1.02, 1.10)	0.008	1.07 (1.02, 1.11)	0.004
Q4 (≥ 1,430)	1,807	46.8	1.09 (1.04, 1.14)	0.001	1.09 (1.04, 1.14)	0.001	1.10 (1.05, 1.17)	0.001
*P* for trend	–	–	–	< 0.001	–	< 0.001	–	< 0.001
**Serum total folate**	–	–	–	–	–	–	–	–
**Quartiles**	–	–	–	–	–	–	–	–
Q1 (< 25.7)	2,489	45.7	Reference	–	Reference	–	Reference	–
Q2 (25.7 < 37.0)	2,430	41.8	0.94 (0.90, 0.98)	0.006	0.95 (0.91, 0.99)	0.021	0.92 (0.88, 0.96)	< 0.001
Q3 (37 < 54.2)	2,337	36.7	0.88 (0.84, 0.92)	< 0.001	0.90 (0.89, 0.94)	< 0.001	0.85 (0.81, 0.89)	< 0.001
Q4 (≥ 54.2)	1,793	33.9	0.85 (0.82, 0.88)	< 0.001	0.87 (0.84, 0.91)	< 0.001	0.77 (0.74, 0.81)	< 0.001
*P*for trend	–	–	–	< 0.001	–	< 0.001	–	< 0.001
**5-mTHF**	–	–	–	–	–	–	–	–
**Quartiles**	–	–	–	–	–	–	–	–
Q1 (< 23.8)	2,491	46.0	Reference	–	Reference	–	Reference	–
Q2 (23.8 < 34.8)	2,390	42.5	0.95 (0.91, 0.99)	0.010	0.95 (0.91,0 .99)	0.021	0.92 (0.88, 0.96)	< 0.001
Q3 (34.8 < 51.2)	2,355	36.2	0.87 (0.83, 0.92)	< 0.001	0.90 (0.86, 0.94)	< 0.001	0.84 (0.80, 0.88)	< 0.001
Q4 (≥ 51.2)	1,786	33.4	0.84 (0.81, 0.87)	< 0.001	0.87 (0.84, 0.90)	< 0.001	0.77 (0.74, 0.80)	< 0.001
*P*for trend	–	–	–	< 0.001	–	< 0.001	–	< 0.001
**UMFA**	–	–	–	–	–	–	–	–
**Quartiles**	–	–	–	–	–	–	–	–
Q1 (< 0.46)	2,458	37.2	Reference	–	Reference	–	Reference	–
Q2 (0.46 < 0.71)	2,570	40.2	0.99 (0.95, 1.03)	0.667	0.99 (0.95, 1.02)	0.458	0.98 (0.94, 1.01)	0.237
Q3 (0.71 < 1.06)	2,117	41.5	1.00 (0.96, 1.05)	0.981	0.99 (0.95, 1.03)	0.489	0.97 (0.93, 1.01)	0.133
Q4 (≥ 1.06)	1,877	41.7	0.97 (0.92, 1.03)	0.335	0.97 (0.92, 1.02)	0.252	0.94 (0.89, 1.00)	0.057
*P* for trend	–	–	–	0.392	–	0.257	–	0.023

^†^Boldface indicates statistical significance (*P* < 0.05).

^1^Crude Model: adjusted for age, sex, ethnicity.

^2^Model 1: adjusted for age, sex, ethnicity, education level, marital status, ratio of family income to poverty (PIR), waist circumference, physical activity status, total energy intake, total sugar intake, total fat intake, food folate intake, smoking, alcohol use, diabetes, hypertension.

^3^Model 2: adjusted for variables in model 1, plus mutually adjustment for the concentration of other folate forms.

**TABLE 3:** Associations between different folate forms and obesity in older participants^†^.

Variables	Total	Events (%)	Crude models^1^		Adjusted models 1^2^		Adjusted models 2^3^	
			**OR (95% CI)**	***P***	**OR (95% CI)**	***P***	**OR (95% CI)**	***P***
**RBC folate**	–	–	–	–	–	–	–	–
**Quartiles**	–	–	–	–	–	–	–	–
Q1 (< 824)	405	35.3	Reference	–	Reference	–	Reference	–
Q2 (824 < 1,090)	501	39.3	1.08 (0.95, 1.22)	0.231	1.07 (0.95, 1.21)	0.265	1.08 (0.96, 1.23)	0.210
Q3 (1,090 < 1,430)	566	36.0	1.03 (0.91, 1.15)	0.672	1.06 (0.95, 1.19)	0.305	1.09 (0.97, 1.22)	0.157
Q4 (≥ 1,430)	1,121	37.5	1.07 (0.96,1.19)	0.247	1.09 (0.98,1.20)	0.117	1.16 (1.04,1.29)	0.014
*P* for trend	–	–	–	0.265	–	0.120	–	0.013
**Serum total folate**	–	–	–	–	–	–	–	–
**Quartiles**	–	–	–	–	–	–	–	–
Q1 (< 25.7)	427	45.9	Reference	–	Reference	–	Reference	–
Q2 (25.7 < 37.0)	466	39.0	1.00 (0.88, 1.12)	0.938	1.02 (0.91, 1.14)	0.758	1.01 (0.90, 1.14)	0.861
Q3 (37 < 54.2)	573	35.7	1.00 (0.89, 1.12)	0.942	1.02 (0.92, 1.13)	0.703	1.00 (0.90, 1.12)	0.943
Q4 (≥ 54.2)	1127	33.8	0.96 (0.87, 1.06)	0.404	1.01 (0.92, 1.11)	0.878	0.97 (0.87, 1.09)	0.632
*P* for trend	–	–	–	0.559	–	0.738	–	0.650
**5-mTHF**	–	–	–	–	–	–	–	–
**Quartiles**	–	–	–	–	–	–	–	–
Q1 (< 23.8)	423	44.9	Reference	–	Reference	–	Reference	–
Q2 (23.8 < 34.8)	484	39.8	1.02 (0.92, 1.14)	0.714	1.04 (0.93, 1.16)	0.485	1.03 (0.92, 1.15)	0.619
Q3 (34.8 < 51.2)	564	35.8	1.03 (0.92, 1.15)	0.638	1.05 (0.95, 1.17)	0.325	1.03 (0.93, 1.15)	0.585
Q4 (≥ 51.2)	1122	33.8	0.97 (0.88, 1.07)	0.547	1.02 (0.93, 1.12)	0.670	0.98 (0.89, 1.09)	0.752
*P* for trend	–	–	–	0.747	–	0.455	–	0.868
**UMFA**	–	–	–	–	–	–	–	–
**Quartiles**	–	–	–	–	–	–	–	–
Q1 (< 0.46)	330	32.7	Reference	–	Reference	–	Reference	–
Q2 (0.46 < 0.71)	549	38.4	1.05 (0.95, 1.17)	0.335	1.05 (0.96, 1.16)	0.294	1.06 (0.96, 1.17)	0.237
Q3 (0.71 < 1.06)	737	38.3	1.03 (0.93, 1.15)	0.554	1.05 (0.96, 1.15)	0.289	1.07 (0.97, 1.17)	0.198
Q4 (≥ 1.06)	977	37.1	1.09 (0.99, 1.19)	0.095	1.09 (1.00, 1.18)	0.055	1.12 (1.03, 1.22)	0.017
*P* for trend	–	–	–	0.178	–	0.080	–	0.034

^†^Boldface indicates statistical significance (*P* < 0.05).

^1^Crue Model: adjusted for age, sex, ethnicity.

^2^Model 1: adjusted for age, sex, ethnicity, education level, marital status, ratio of family income to poverty (PIR), waist circumference, physical activity status, total energy intake, total sugar intake, total fat intake, food folate intake, smoking, alcohol use, diabetes, hypertension.

^3^Model 2: adjusted for variables in model 1, plus mutually adjustment for the concentration of other folate forms.

However, a significant negative association was found between serum total folate levels and risk of obesity, with ORs of 0.929 (95% CI: 0.893–0.968), 0.870 (95% CI: 0.835–0.907), 0.804 (95% CI: 0.773–0.835) observed in Q2, Q3, and Q4 groups, respectively (quartile trend *P* < 0.001 in adjusted Model 2) (Supplementary Table 1). Meanwhile, a notable negative correlation between serum 5-mTHF levels and obesity risk was identified, with ORs of 0.930 (95% CI: 0.895–0.966), 0.866 (95% CI: 0.829–0.903), and 0.800 (95% CI: 0.772–0.830) observed in the Q2, Q3, and Q4 groups, respectively (quartile trend *P* < 0.001) (Supplementary Table 1). This trend majorly persisted among middle-aged participants (quartile trend *P* < 0.001) (Table 2), with the lowest risk of obesity among middle-aged adults in the top quartile (Q4) group for serum total folate and serum 5-mTHF. However, this association was absent in the older participants (Table 3).

No significant association between serum UMFA and obesity risk was found in total participants (Supplementary Table 1) and the middle-aged group (Table 2) after adjusting for covariates (Model 2). Notably, older participants with UMFA levels in the fourth quartile (≥ 1.06 nmol/L) had a higher risk of obesity (OR, 1.056; 95% CI: 1.004–1.110) compared with those with lower UMFA levels (Table 3).

### 3.3 Stratified analyses

**FIGURE 1:**
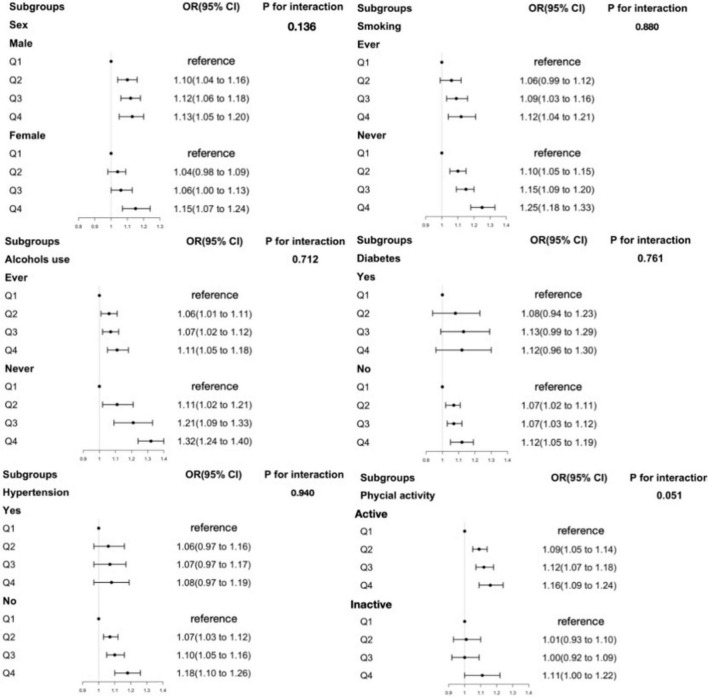
The association between RBC folate and obesity in middle-aged participants in subgroups. Adjusted for age, sex, ethnicity, education level, marital status, ratio of family income to poverty (PIR), waist circumference, physical activity status, total energy intake, total sugar intake, total fat intake, food folate intake, smoking, alcohol use, diabetes, hypertension. RBC folate, red blood cell folate.

**FIGURE 2:**
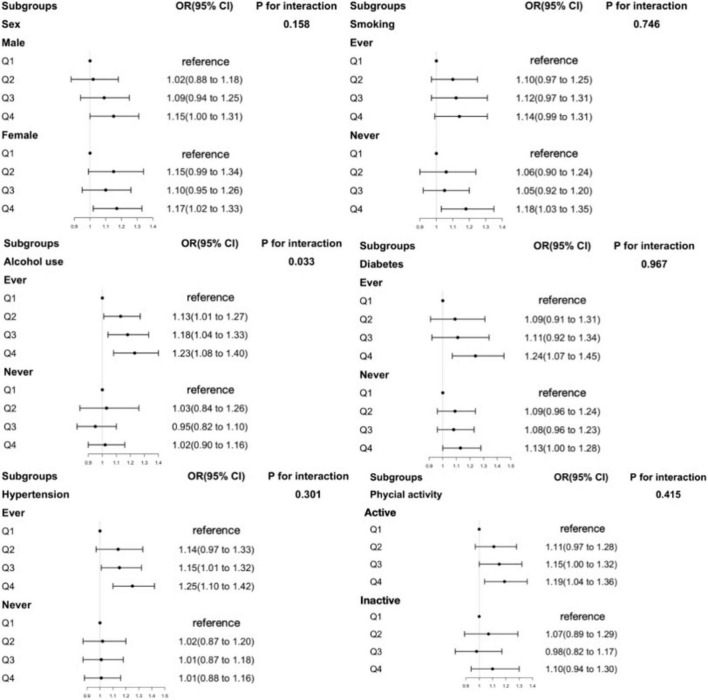
The association between RBC folate and obesity in older participants in subgroups. Adjusted for age, sex, ethnicity, education level, marital status, ratio of family income to poverty (PIR), physical activity status, total energy intake, total sugar intake, total fat intake, food folate intake, smoking, alcohol use, diabetes, hypertension. RBC folate, red blood cell folate.

Stratified analyses (Figures 1, 2 and Supplementary Figures 2–5) evaluating the associations of obesity risk with RBC folate, 5-mTHF, or UMFA showed no significant modification in the relationships between folate forms and obesity risk (*P* for interactions > 0.05). And the folate-obesity associations were not modified by smoking, alcohol use, diabetes, hypertension, and physical activity in the current study (all *P* for interactions > 0.05) (Figures 1, 2 and Supplementary Figures 2–5). In addition, we compared folate levels between overweight and obese individuals in middle-aged and older participants. The results showed that in middle-aged participants, the obese group had significantly higher RBC folate levels than the overweight group (*P* < 0.05), while serum total folate and 5-mTHF levels were significantly lower in the obese group compared to the overweight group (*P* < 0.001). In contrast, among older participants, there were no significant differences in the levels of various folate forms between the overweight and obese groups (*P* > 0.05) (Supplementary Table 2).

## 4 Discussion

In a representative United States adult sample, we first revealed an age-specific association between serum folate forms and obesity risk. Our study confirmed that increased RBC folate levels are associated with a higher risk of obesity. Moreover, we observed that higher serum 5-mTHF levels decreased obesity risk in the middle-aged participants, while UMFA levels had no significant effect. In contrast, higher levels of UMFA were associated with an increased risk of obesity in older participants.

Past studies have linked RBC folate and serum total folate levels with obesity, showing that RBC folate levels are higher in obese individuals, whereas dietary folate intake is negatively correlated with overweight and obesity (16, 17). Consistent with these findings, our study identified a positive correlation between RBC folate levels and obesity risk, while serum total folate and serum 5-mTHF levels were negatively associated with obesity risk. Notably, we observed an age-related difference in the protective effects of increased serum total folate and serum 5-mTHF levels against obesity. These associations were significant in middle-aged participants but absent in older individuals, likely reflecting age-related physiological and metabolic changes. Dietary habits and lifestyle factors may also contribute to these age-dependent differences, further influencing the relationship between serum total folate, 5-mTHF, and obesity. Our findings underscore the importance of maintaining adequate folate levels, particularly in middle-aged individuals, to reduce the risk of obesity.

The association between low folate levels and obesity observed in this study can be interpreted in two ways. Specifically, on the one hand, low folate levels may contribute to the development of obesity; on the other hand, obesity may affect folate metabolism and utilization. Firstly, insufficient folate levels may lead to elevated homocysteine (Hcy) concentrations, which in turn promote the development of obesity. Previous studies have demonstrated that individuals with obesity generally exhibit higher plasma Hcy levels compared to those with normal body weight (18, 19). A study by Asemi et al. revealed that folate supplementation can significantly reduce plasma Hcy concentrations and improve serum insulin, total cholesterol, low-density lipoprotein cholesterol (LDL-C), high-density lipoprotein cholesterol (HDL-C), and the homeostasis model assessment of insulin resistance (HOMA-IR). In addition, folate deficiency may suppress the phosphatidylinositol 3-kinase (PI3K) signaling pathway, thereby exacerbating insulin resistance and further impairing lipid metabolism (20). Folate also serves as a critical cofactor in one-carbon metabolism, which plays a key role in DNA methylation. Folate deficiency-induced aberrant DNA methylation has broader implications for metabolic dysregulation, including impaired glycemic control in diabetes and hepatic lipid accumulation in non-alcoholic fatty liver disease (21). Such epigenetic disturbances can also disrupt energy and lipid metabolism, ultimately increasing the risk of obesity (22–26). Previous research has indicated a correlation between hypomethylation status and increased body weight, particularly among women of reproductive age (27). Moreover, methylenetetrahydrofolate reductase (MTHFR) is a key enzyme that catalyzes the conversion of 5,10-methylenetetrahydrofolate to 5-methyltetrahydrofolate. The C677T polymorphism in the MTHFR gene, especially the TT genotype, has been associated with reduced folate levels and a higher risk of obesity (28–30). Notably, this polymorphism is also linked to insulin resistance and NAFLD progression, potentially through disrupted homocysteine metabolism and methylation capacity (31, 32). On the other hand, low serum folate levels may also be a consequence of obesity. Metabolic alterations induced by obesity may impair folate utilization and increase the individual requirement for folate. Several studies have shown that despite comparable folate intake, individuals with obesity tend to have significantly lower serum folate concentrations than those with normal body weight (3, 33, 34). Interestingly, it has also been observed that while fasting serum folate levels are lower in individuals with obesity, their RBC folate concentrations are paradoxically higher (4, 35), which may reflect a compensatory mechanism whereby decreased serum folate levels stimulate enhanced folate uptake by RBCs. Mechanistic studies suggest that obesity is associated with increased activity of cytochrome P450 2E1 (CYP2E1), an enzyme that metabolizes folate as a substrate (36). Therefore, enhanced folate degradation mediated by CYP2E1 may be one of the mechanisms contributing to reduced serum folate levels in obese individuals. Additionally, the accumulation of adipose tissue in obesity may elevate circulating estrogen levels, which has also been proposed as a contributing factor to folate deficiency (37).

Folic acid, commonly used in supplements and food fortification, demonstrates limited reduction in the human gut and methylation in the liver, leading to unmetabolized folic acid (UMFA) in circulation when consumed excessively (38). Numerous studies have associated folic acid intake with non-cancer health outcomes, particularly metabolic diseases. For instance, UMFA has been linked to an increased risk of gestational diabetes mellitus (GDM) in Chinese populations (39, 40). Animal studies further demonstrate that excessive perinatal folic acid supplementation induces insulin resistance, dyslipidemia, and disruptions in glucose and hepatic fat metabolism in both mice (41) and rat offspring (42–44). In line with these findings, our study revealed a positive association between high serum UMFA concentrations and obesity in older adults, while no such link was observed in middle-aged individuals. Potential mechanisms underlying this relationship include UMFA-induced disruptions in DNA and protein methylation, resulting in abnormal gene expression associated with obesity. These disruptions may impair the functionality of proteins critical for metabolic regulation, influencing lipid synthesis, breakdown, and energy balance, ultimately leading to increased fat accumulation and obesity risk. An-other plausible explanation is the disruption of intracellular folate metabolism. Accumulated UMFA, closely associated with the saturation of dihydrofolate reductase (DHFR), may inhibit DHFR activity in a competitive or non-competitive manner, depending on intracellular dihydrofolate (DHF) concentrations—a key intermediate in the thymidylate synthesis pathway (45). This inhibition could result in DHF accumulation, which is a potent inhibitor of methylenetetrahydrofolate reductase (MTHFR), thereby disrupting the one-carbon cycle (46–48). Such disruptions can impair essential cellular processes, including DNA synthesis, repair, and methylation, particularly in older adults who experience a decline in folic acid conversion efficiency (49).

When exploring the age-specific associations between folate and obesity risk, we systematically adjusted for potential confounding factors such as smoking, alcohol use, diabetes, hypertension, and physical activity. The results showed that even after incorporating these important confounding factors into the adjustment, the association between folate and obesity remained stable and did not change significantly. This finding preliminarily indicates that folate may play a role in pathways such as adipocyte metabolism and DNA methylation, and it is less likely to be influenced by external factors like smoking, alcohol use, diabetes, hypertension, or physical activity, indicating a potentially close and stable underlying associations between folate and obesity. In addition to the factors we have investigated, the impact of environmental factors on obesity should not be overlooked. Environmental exposure to different compounds may increase the risk of obesity. Pollutants in the environment, such as persistent organic pollutants (POPs) and heavy metals, may disrupt the human endocrine system, affect hormonal balance, and thereby alter the mechanisms of fat metabolism and energy regulation (50). Considering that obesity is a complex disease caused by multiple factors, it is crucial to adopt a multi-factor approach for prospective obesity treatment. This means that when formulating obesity treatment strategies, we should not only focus on an individual’s nutritional intake and metabolic status but also take environmental factors into account. By comprehensively evaluating various factors such as environmental exposure, lifestyle, genetic factors, and metabolic characteristics, we can gain a more comprehensive understanding of the pathogenesis of obesity. This will enable us to develop more targeted and effective intervention measures, opening new avenues for the prevention and treatment of obesity.

From a public health perspective, it is crucial to continuously monitor the folate status of the United States population, particularly among older adults, to ensure the safety of folic acid fortification programs (51, 52). In terms of nutritional intervention, we believe it is essential to pay particular attention to the absorption and conversion capacity of folic acid in older individuals, aiming to reduce the levels of UMFA, thus potentially lowering the risk of obesity among the older population. Given the essential role of folate in one-carbon metabolism and DNA methylation, we recommend meeting the nutritional needs of older adults by directly supplementing with active folate forms. This approach may help maintain adequate folate levels while contributing to a reduction in obesity risk.

This study has several notable strengths, including a nationally representative large sample size, standardized measurement protocols, and detailed data on various serum folate forms. The use of comprehensive adjustments for potential confounders allowed for a more robust investigation into the associations between RBC folate, different serum folate forms, and obesity. Moreover, the application of NHANES sampling weights enhances the generalizability of the findings to the broader United States population. However, our analysis also has some limitations to be addressed. First, the cross-sectional nature of the study only provided association clues and limited causative inferences and generalization to other populations. Second, due to the lack of available data in the selected NHANES cycles, it was not possible to account for the potential influence of vitamin B12, vitamin B6, S-adenosylmethionine (SAM), and homocysteine levels when exploring the relationship between folate status and obesity. This may have limited a more comprehensive understanding of the underlying mechanisms. It remains unclear whether folate status in obesity is a causal factor or a biomarker response to weight changes. Given folate’s role in one-carbon metabolism and mandatory fortification policies, further research is crucial for deeper insights.

## 5 Conclusion

In conclusion, using a large, representative data of United States adults, we found that there were significant positive relationships of RBC folate with the risk of obesity. Additionally, our findings indicate a nuanced relationship showing low serum 5-mTHF levels associated with a higher risk of obesity among the middle-aged adults. Conversely, in older participants, elevated serum UMFA levels are associated with a higher risk of obesity. Given prevalent folic acid fortification and supplementation use, our findings underscore the need for monitoring folate form concentrations, potentially guiding future clinical trials on folate supplementation.

## Data Availability

The raw data supporting the conclusions of this article will be made available by the authors, without undue reservation.

## Footnotes

1
http://www.R-project.org/
