# Supplementary material for: Age-specific associations of RBC folate and several serum folate forms with obesity risk: NHANES 2011–2018
Source: Front Nutr. 2025 Apr 10;12:1547844. doi: 10.3389/fnut.2025.1547844 (PMC12020389; doi:10.3389/fnut.2025.1547844)
Supplement: Supplementary file 1 [file Data_Sheet_1.zip › Supplementary Files/Figure S4.docx]

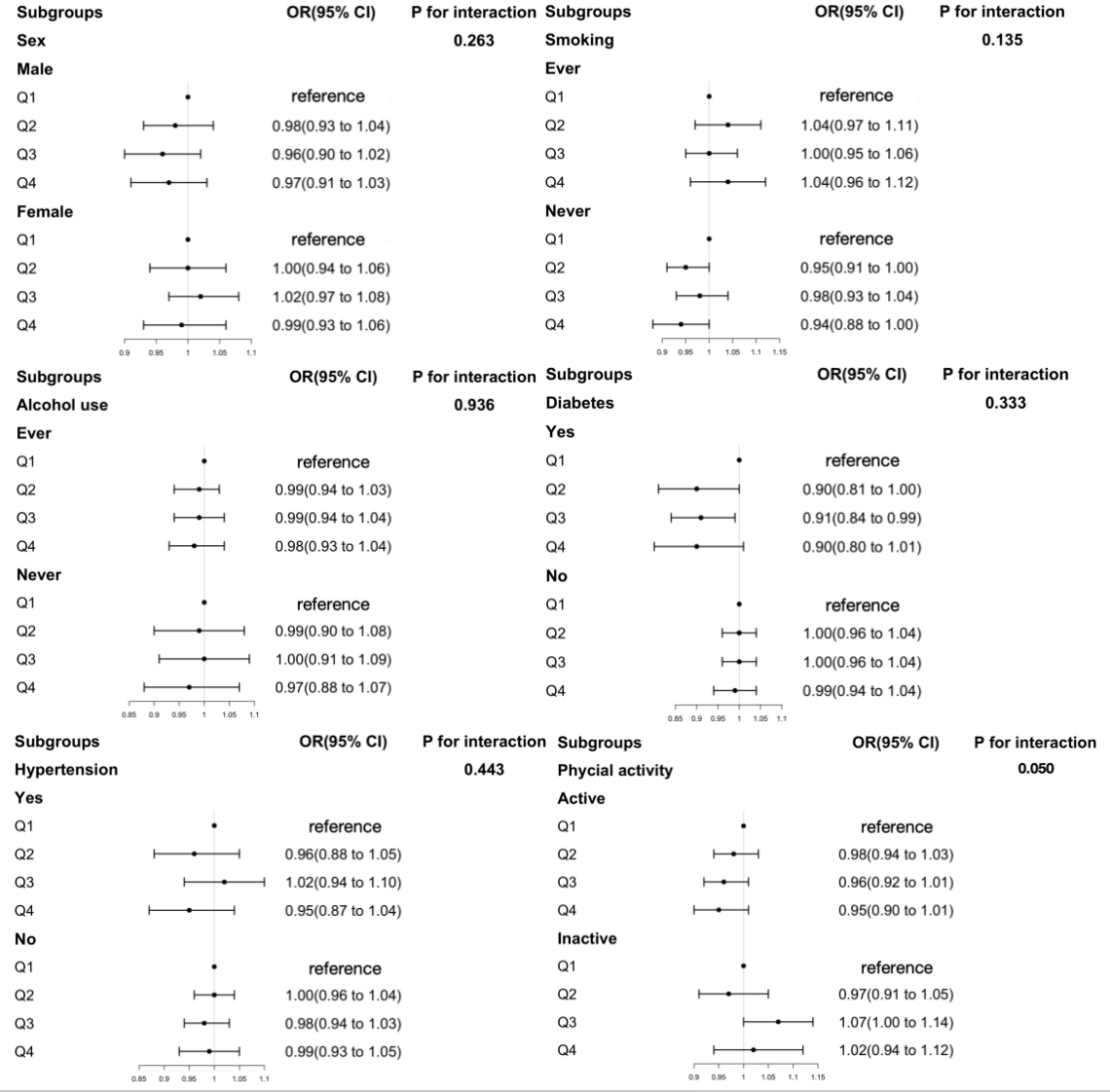


**Figure S4.** The association between UMFA and obesity in middle-aged participants in subgroups. Adjusted for age, sex, ethnicity, education level, marital status, PIR, physical activity status, total energy intake, total sugar intake, total fat intake, smoking, alcohol use, diabetes, hypertension. Abbreviations: UMFA, unmetabolized folic acid.
