# Supplementary material for: Age-specific associations of RBC folate and several serum folate forms with obesity risk: NHANES 2011–2018
Source: Front Nutr. 2025 Apr 10;12:1547844. doi: 10.3389/fnut.2025.1547844 (PMC12020389; doi:10.3389/fnut.2025.1547844)
Supplement: Supplementary file 1 [file Data_Sheet_1.zip › Supplementary Files/Table S2.docx]

**Table S2.** **Folate status in overweight and obese adults in middle-aged participants and older participants**.†

| **Characteristics** | **Middle-aged participants** | | | **Older participants** | | | |
| --- | --- | --- | --- | --- | --- | --- | --- |
|  | **Overweight, N=2785 ^1^** | **Obesity, N=3613^1^** | ***P*^2^** | | **Overweight, N=979 ^1^** | **Obesity, N=965^1^** | ***P*^2^** |
| RBC folate (nmol/L) | 1110.0  (853.0, 1390.0) | 1120.0  (876.0, 1460.0) | 0.042 | | 1410.0  (1000, 1840) | 1420.0  (1010.0, 1870.0) | 0.999 |
| Serum Total Folate (nmol/L) | 38.0  (26.6, 52.5) | 34.0  (23.7, 47.8) | <0.001 | | 50.0  (32.9, 79.3) | 48.4  (30.6, 72.0) | 0.167 |
| 5-mTHF (nmol/L) | 35.8  (24.9, 49.1) | 32.0  (21.9, 45.2) | <0.001 | | 48.5  (30.8, 73.7) | 45.3  (28.1, 67.0) | 0.153 |
| UMFA (nmol/L) | 0.67  (0.50, 1.00) | 0.67  (0.49, 0.98) | 0.684 | | 0.84  (0.59, 1.43) | 0.96  (0.62, 1.54) | 0.148 |

†Boldface indicates statistical significance (*P*<0.05)

^1^ Continuous values are given as the median (the 25% and 75% quartiles).

^2^ chi-squared test with Rao & Scott’s second-order correction.

Abbreviations: RBC folate, red blood cell folate; 5-mTHF, 5-methylenetetrahydrofolate; UMFA, unmetabolized folic acid.
