# Supplementary material for: Age-specific associations of RBC folate and several serum folate forms with obesity risk: NHANES 2011–2018
Source: Front Nutr. 2025 Apr 10;12:1547844. doi: 10.3389/fnut.2025.1547844 (PMC12020389; doi:10.3389/fnut.2025.1547844)
Supplement: Supplementary file 1 [file Data_Sheet_1.zip › Supplementary Files/Table S1.docx]

**Table S1.** Associations between different folate forms and obesity in total participants. †

|  | **Total** | **Events (%)** | **Crude Models** ^1^ | | **Adjusted Models 1** ^2^ | | **Adjusted Models 2** ^3^ | |
| --- | --- | --- | --- | --- | --- | --- | --- | --- |
|  |  |  | **OR (95% CI)** | ***P*** | **OR (95% CI)** | ***P*** | **OR (95% CI)** | ***P*** |
| **RBC Folate** |  |  |  |  |  |  |  |  |
| **Quartiles** |  |  |  |  |  |  |  |  |
| Q1(<824) | 2894 | 35.5 | reference |  | reference |  | reference |  |
| Q2(824-<1090) | 2931 | 39.5 | 1.04 (1.00,1.08) | 0.076 | 1.05 (1.01,1.09) | 0.013 | 1.05 (1.02,1.09) | 0.010 |
| Q3(1090-< 1430) | 2862 | 39.2 | 1.03 (0.99,1.07) | 0.188 | 1.06 (1.02,1.10) | 0.005 | 1.07 (1.03,1.12) | 0.003 |
| Q4(≥1430) | 2928 | 43.2 | 1.07 (1.03,1.12) | 0.003 | 1.08 (1.03,1.13) | 0.001 | 1.11 (1.04,1.18) | 0.003 |
| *P* for trend |  |  |  | <0.001 |  | <0.001 |  | 0.001 |
| **Serum Total Folate** |  |  |  |  |  |  |  |  |
| **Quartiles** |  |  |  |  |  |  |  |  |
| Q1(<25.7) | 2916 | 45.7 | reference |  | reference |  | reference |  |
| Q2(25.7-<37.0) | 2869 | 41.4 | 0.94 (0.91,0.98) | 0.009 | 0.95 (0.92,0.99) | 0.028 | 0.93 (0.89,0.97) | 0.001 |
| Q3(37-<54.2) | 2910 | 36.5 | 0.89 (0.85,0.93) | <0.001 | 0.91 (0.88,0.95) | 0.001 | 0.87 (0.84,0.91) | <0.001 |
| Q4(≥54.2) | 2920 | 33.9 | 0.86 (0.83,0.88) | <0.001 | 0.89 (0.86,0.92) | <0.001 | 0.80 (0.77,0.84) | <0.001 |
| *P* for trend |  |  |  | <0.001 |  | <0.001 |  | <0.001 |
| **5-mTHF** |  |  |  |  |  |  |  |  |
| **Quartiles** |  |  |  |  |  |  |  |  |
| Q1(<23.8) | 2914 | 45.8 | reference |  | reference |  | reference |  |
| Q2(23.8-<34.8) | 2874 | 42.0 | 0.95 (0.91,0.99) | 0.010 | 0.96 (0.92,0.99) | 0.024 | 0.93 (0.90,0.97) | <0.001 |
| Q3(34.8-<51.2) | 2919 | 36.1 | 0.89 (0.85,0.93) | <0.001 | 0.91 (0.87,0.95) | <0.001 | 0.87 (0.83,0.90) | <0.001 |
| Q4(≥51.2) | 2908 | 33.6 | 0.85 (0.83,0.88) | <0.001 | 0.88 (0.85,0.91) | <0.001 | 0.80 (0.77,0.83) | <0.001 |
| *P* for trend |  |  |  | <0.001 |  | <0.001 |  | <0.001 |
| **UMFA** |  |  |  |  |  |  |  |  |
| **Quartiles** |  |  |  |  |  |  |  |  |
| Q1(<0.46) | 2788 | 36.7 | reference |  | reference |  | reference |  |
| Q2(0.46-<0.71) | 3119 | 39.9 | 0.99 (0.96,1.04) | 0.787 | 0.99 (0.96,1.03) | 0.707 | 0.99 (0.95,1.03) | 0.527 |
| Q3(0.71-<1.06) | 2854 | 40.7 | 1.00 (0.99,1.04) | 0.817 | 0.99 (0.96,1.03) | 0.702 | 0.98 (0.94,1.02) | 0.370 |
| Q4(≥1.06) | 2854 | 40.1 | 0.98 (0.94,1.03) | 0.443 | 0.98 (0.94,1.03) | 0.427 | 0.96 (0.92,1.01) | 0.134 |
| *P* for trend |  |  |  | 0.427 |  | 0.338 |  | 0.059 |

†Boldface indicates statistical significance (*P*<0.05)

^1^ Crude Model: adjusted for age, sex, ethnicity.

^2^ Model 1: adjusted for age, sex, ethnicity, education level, marital status, PIR, waist circumference, physical activity status, total energy intake, total sugar intake, total fat intake, smoking, alcohol use, diabetes, hypertension.

^3^ Model 2: adjusted for variables in model 1, plus mutually adjustment for the concentration of other folate forms.
