# Supplementary figures and images for: Age-specific associations of RBC folate and several serum folate forms with obesity risk: NHANES 2011–2018
Source: Front Nutr. 2025 Apr 10;12:1547844. doi: 10.3389/fnut.2025.1547844 (PMC12020389; doi:10.3389/fnut.2025.1547844)

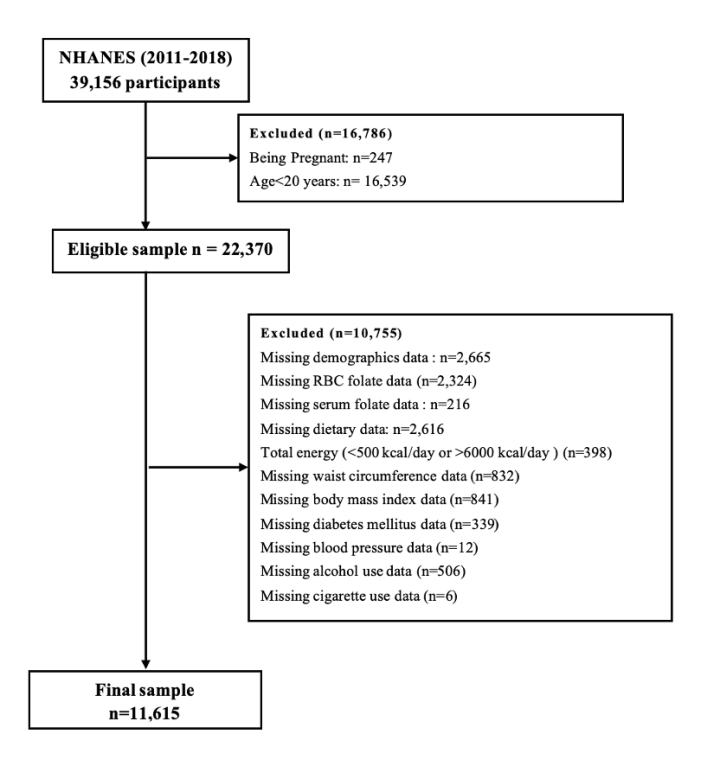


**Figure S1.** Flow chart of the participants in the current analysis.

Supplement: Supplementary file 1 [file Data_Sheet_1.zip › Supplementary Files/Figure S1.docx]
